# Deficit of circulating stem – progenitor cells in opiate addiction: a pilot study

**DOI:** 10.1186/1747-597X-2-19

**Published:** 2007-07-05

**Authors:** Albert S Reece, Peter Davidson

**Affiliations:** 1 Southcity Family Medical Centre, 39 Gladstone Rd., Highgate Hill, Brisbane, Queensland, 4101, Australia; 2 Queensland Medical Laboratories Pathology, P.O. Box 2280, Mansfield QLD, 4122, Australia

## Abstract

A substantial literature describes the capacity of all addictive drugs to slow cell growth and potentiate apoptosis. Flow cytometry was used as a means to compare two lineages of circulating progenitor cells in addicted patients. Buprenorphine treated opiate addicts were compared with medical patients. Peripheral venous blood CD34^+ ^CD45^+ ^double positive cells were counted as haemopoietic stem cells (HSC's), and CD34^+ ^KDR^+ ^(VEGFR2^+^) cells were taken as endothelial progenitor cells (EPC's). 10 opiate dependent patients with substance use disorder (SUD) and 11 non-addicted (N-SUD) were studied. The ages were (mean + S.D.) 36.2 + 8.6 and 56.4 + 18.6 respectively (P <0.01). HSC's were not different in the SUD (2.38 + 1.09 Vs. 3.40 + 4.56 cells/mcl). EPC's were however significantly lower in the SUD (0.09 + 0.14 Vs. 0.26 + 0.20 cells/mcl; No. > 0.15, OR = 0.09, 95% C.I. 0.01–0.97), a finding of some interest given the substantially older age of the N-SUD group. These laboratory data are thus consistent with clinical data suggesting accelerated ageing in addicted humans and implicate the important stem cell pool in both addiction toxicology and ageing. They carry important policy implications for understanding the fundamental toxicology of addiction, and suggest that the toxicity both of addiction itself and of indefinite agonist maintenance therapies may have been seriously underestimated.

## Background

A significant literature describes the capacity of all addictive drugs to slow cell growth [1-3] on the one hand and to potentiate apoptosis[4-8] on the other. The stem cell hypothesis of ageing suggests that ageing at the organismal level is reflected by impaired cell health at the cellular level including reduced function, reduced growth, increased senescence, and cell loss by apoptosis, necrosis and other pathways[9,10]. The often disorganized and disheveled body habitus of many drug addicts is well known as is their predisposition to a variety of unusual disorders. Indeed the addiction literature features a variety of disorders well known to occur in geriatric populations[11]. Data from our clinic and elsewhere suggests that disorders common in aged populations occur at an increased frequency in addicted populations. This applies to osteoporosis[12,13] neuropsychiatric disorders[14-16] depressed sperm counts[17,18] calcific arteriosclerosis[19] graying of the hair[20], and severe dental disorders[21,22]. These problems have in common a failure in stem cell physiology. The high mortality accompanying chemical addictions is also well recognized[23,24].

The field of stem cell biology of course is burgeoning with many investigations centred around a host of prospective applications in regenerative medicine and including particularly bone marrow transplantation, tissue regeneration and immune and gene therapy. Several recent developments make these advances of particular interest to the field of addictive medicine particularly to its toxicology. Stem cells exist at low frequency in the peripheral circulating blood and may be quantitated there. Several methods have been recently described for the quantitation of various lines of stem and progenitor cells in peripheral blood[25-27] a tissue which is regularly accessed in routine clinical care. Secondly the endothelial progenitor cell has been said to be of enormous importance to the regeneration of the vasculature and has be noted to be a superior predictor of cardiovascular outcomes including mortality than commonly used classical cardiovascular risk factors[25]. Similarly counts of the circulating osteoblastic progenitor cell has been shown to correlate with bone density studies[27]. Finally the cellular theory of ageing suggests that stem cells and their health should be a special focus of ageing medicine and the deficits associated with ageing, and this has been confirmed by recent reports[28,29].

Therefore it seemed important to us examine the peripheral blood for circulating stem cell numbers in addicted and control populations. The ability to quantitate many different lines of tissue specific stem cells invites an opportunity to quantitate any supposed progeroid (pro-ageing) effect in a variety of tissues by relatively straightforward technical means. As our clinic sees both addicts and non-addicts in a primary care setting this population seemed to provide an ideal opportunity to test the hypothesis. The present results were reported when to our great surprise statistical significance and interesting results were obtained in the initial pilot study with only two lines of progenitor cells and very limited patient numbers.

Whilst the present report is clearly of a preliminary nature, should its main findings be confirmed by more sophisticated laboratory and clinical studies the implications are potentially far reaching indeed. In suggesting that the toxicity of addiction has been in large measure understated, it implies that the drug policy debate might be potentially re-cast by emerging evidence; in suggesting that addictive agents have a deleterious effect on cell growth and regeneration it implies that indiscriminate application of indefinite agonist therapies should be re-prioritized downwards in treatment protocols; in noting that there are indications that the integrity of DNA replication is likely compromised by addiction it informs studies of tumour development; and in directly implicating the classical receptor-ligand pathways of addiction in the biology of ageing it suggests new pathways of investigation and identification of drug targets for the treatment of classical age related degenerative pathologies.

## Methods

### Patients

Patients were chosen from our normal clinical primary care population. Medical patients were representative of those seen typically in primary care clinics. Opiate addicted patients are maintained on buprenorphine/naloxone combination and are gradually reduced. They are not in clinical withdrawal at any time. Blood was obtained with patient consent. Blood was drawn for standard clinical indications in the course of routine patient care. Peripheral venous blood was sampled from patients and processed fresh without storage by flow cytometry. Absolute lymphocyte counts were taken, and CD34^+ ^CD45^+ ^double positive cells were counted as haemopoietic stem cells (HSC's), and CD34^+ ^KDR^+ ^(VEGFR2^+^) cells were denoted endothelial progenitor cells (EPC's). Progenitor cells were standardized against the lymphocyte fraction as these were believed to be of the most appropriate nuclear cytoplasmic ratio.

### Flow cytometry

0.5 ml Peripheral blood collected in EDTA TUBES (BD). The samples were prepared for flow cytometry analysis using the Coulter TQ-Prep. The samples were incubated with the monoclonal antibodies – CD34 FITC (BD Cat No. 348053);CD45 PC5 (ID Test PN IM2652); KDR anti-hVEGF PE (R&D Systems Cat No. FAB 357P). 20 mcl of antibodies were used in accordance with the manufacturer's instructions. Samples were run on the Beckman Coulter EPICs XL-MCL flow cytometer. Histograms using forward scatter/side scatter; CD45vCD34; CD34vKDR were generated. The gating strategy employed was as follows. 3 histograms were set up. The first was CD45 V's side scatter linear a rectilinear region (region A) set to include all CD45 positive events. The second, CD34 V's side scatter linear – was gated on region A. A rectilinear region (region B) was set to include all CD34 positive events. The third region CD34 V's KDR- was gated on A and B. A quadstat region was set up and the dual CD34/KDR population reported.

The percentage of CD34+/KDR+ and CD34+/KDR- cells was quantitated. 10,000 events were counted per sample.

### Statistical Analysis

Categorical data were analyzed by the 2 tailed Fisher Exact test. Continuous data was analyzed by the Student's T-test where normally distributed. These results were verified by non-parametric analyses such as Friedman ANOVA. On occasion more sophisticated multiple regression techniques were required as detailed in the text. P less than 0.05 was considered significant.

### Ethical Approval

This study was approved by the Institutional review board of the Southcity Medical Centre, which is a Human Ethics Research Committee registered with the National Health And Medical Research Council of Australia (No. 000409).

## Results

### Patient characterization

Patient demographic and medical characteristics are shown in Table 1. 10 opiate addicted SUD and 11 non-addicted (N-SUD) were studied. N-SUD were considerably older than SUD (means + S.D. 36.20 + 8.61 and 56.36 + 18.56 Student's T = 3.13, df = 19, P = 0.0054). All SUD were male and 55% of N-SUD were male (Fisher Exact test P = 0.035). All patients were of European – Australian ethnic background. In keeping with the younger age of the SUD, this group was taller (179.30 + 11.61 Vs. 169.82 + 8.22 Student's T = -2.177, df = 19, P = 0.042283). As shown the weight, BMI, systolic and diastolic blood pressures were not different. Two patients in each group (20% and 18%) had a remote history of cardiovascular disease 2–10 years before (sub-acute bacterial endocarditis and insulin dependent diabetes mellitus; and multiple pulmonary embolus and coronary heart disease, myocardial infarction and hypertension respectively). The last mentioned control patient was currently taking cardiovascular medication.

**Table 1:** Demographic data

**Variable**	**SUD**	**Non-SUD**	P*
**Age**	36.20 (8.61)	56.36 (18.56)	0.00543
**% Male**	100%	55%	0.03508
**Height**	179.30 (11.61)	169.82 (8.22)	0.04228
**Weight**	76.70 (13.53)	75.64 (18.16)	0.88003
**BMI**	23.77 (2.79)	26.00 (4.47)	0.18506
**BP Systolic**	115.00 (9.72)	119.67 (14.76)	0.43506
**BP Diastolic**	70.50 (5.99)	74.44 (11.30)	0.36820
**Vasculopathy**	20%	18%	1.00000

Data as Mean (+ S.D.).

Statistical Tests – Student's T-test for continuous variables;

Fisher Exact test (2 tailed) for categorical variables

### Drug use

The drugs used by this group are described in Table 2. The major drug of addiction for which treatment was sought was opiates (heroin and morphine), but a variety of other agents were commonly used by this cohort particularly tobacco and cannabis and some stimulants as has been previously reported^21^. As shown in the table, the SUD group used significantly more tobacco, cannabis, amphetamine and heroin than N-SUD. The alcohol use rate was not different, and none of the patients in either group had a history of problematic alcohol consumption. Opiate agonist pharmacotherapy was with combination buprenorphine/naloxone. The mean dose of buprenorphine (+ S.D.) was 10.32 + 7.89 mg/d.

**Table 2:** Drug use data

	**SUD**	**Non-SUD**	P*	**O.R. (95%C.I.)/(Details)**
**CATEGORICAL DATA – RELATIVE FREQUENCIES**				
**Tobacco Use**	80%	18%	0.01431	18.00 (1.50–265.16)
**Alcohol Use**	0%	27%	0.83270	0.00 (0.00–2.52)
**Cannabis Use**	90%	18%	0.00815	40.50 (2.36–1963.44)
**Amphetamine Use**	90%	18%	0.00815	40.50 (2.36–1963.44)
**Heroin**	100%	0%	0.00157	Not Defined
**Methadone Use**	30%	9%	0.3107	4.29 (0.26–247.01)
				
**QUANTITATIVE USE DATA**				
**Cigarettes (/day)**	14.70 (10.21)	3.64 (8.09)	0.00468	(df = 19; T = -2.7657)
**Heroin Dose (g/day)**	0.55 (0.34)	0.07 (0.16)	0.00157	(df = 19; T = -5.3803)
**Duration Opiate Addiction (Yrs)**	12.70 (7.10)	3.55 (8.41)	0.01141	(df = 19; T = -2.6805)

Data as Mean (+ S.D.).

* – Statistical Tests – Student's T-test for continuous variables;

Fisher Exact test (2 tailed) for categorical variables

### Laboratory parameters

Table 3 lists the laboratory parameters of the two groups. 90% vs. 18% had serological evidence of previous Hepatitis C infection (Fisher Exact Test P = 0.00190, O.R. = 40.50, 95% C.I. 2.36–1963.44). There was however no difference in the rate of abnormal alanine aminotransferase (ALT; 40% vs. 18% Fisher Exact Test = 0.361) nor its mean value (see Table 3 61.30 + 61.20 vs. 31.33 + 31.28 df = 17, Student's T = -1.32, P = 0.20). No patients were HIV positive.

**Table 3:** Laboratory parameters

	**SUD**	**Non-SUD**	P*
**Serolgy**			
**HCV+**	90%	18%	0.00190
**HIV +**	0%	0%	-
			
**Biochemistry**			
**ALT (Iu/l)**	61.30 (61.20)	31.33 (31.28)	0.20451
**RAISED ALT**	40%	18%	0.36145
**Iron (mcmol/l)**	40.9 (2.69)	45.22 (2.82)	0.03711
**Albumin (/l)**	40.90 (2.69)	45.22 (2.82)	0.00325
**Cholestrol(mmol/l)**	4.26 (1.09)	5.51 (0.95)	0.01685
**Triglyceride (mmol/l)**	1.40 (0.63)	1.40 (.57)	1.00000
**BSL (mmol/l)**	5.63(2.22)	4.57 (0.80)	0.18239
			
**Haematology**			
**Haemoglobin(g/l)1**	140.9(11.96	148.78(12.38)	
**MCV(fl)**	87.00 (2.58)	92.33 (2.96)	0.00061
**PP (x10**^9^**/l)**	258.10 (77.44)	286.89 (79.57)	0.43637
**WCC (x10**^9^**/l)**	6.58 (2.42)	7.39 (2.12)	0.44777
**Lymphocytes (x10**^9^**/l)**	2.23 (0.67)	2.12 (0.78)	0.72715
**Monocytes (x10**^9^**/l)**	0.53 (0.24)	0.59 (0.26)	0.61419
**Mononuclear Cells (x10**^9^**/l)**	2.76 (0.86)	2.18 (1.43)	0.27302
**Neutrophils (x10**^9^**/l)**	3.58 (1.78)	4.41 (1.40)	0.27079
**CD34+/CD45+ (x10**^11^**/l)**	2.38 (1.09)	3.40(4.56)	0.10166
**CD34+/KDR+ (x10**^11^**/l)**	0.09 (0.14)	0.26 (0.20)	0.03674

Data as Mean (+ S.D.)

Statistical Tests – Student's T-test for continuous variables;

Fisher Exact test (2 tailed) for categorical variables

It was of interest that the serum iron (40.9+2.69 vs. 45.22+2.82 Student's T = 2.26, df = 17, P = 0.037) and the mean corpuscular volume (87.00+2.58 vs. 92.33+2.96 fl Student's T = 4.19, df = 17, P = 0.00061) were lower in the SUD group, despite the higher rate of females in the N-SUD group. The five females in the control group were all post-menopausal (mean ages 63.2+15.1 years). The albumin (40.90+2.69 vs. 45.22+2.82 Student's T = 3.42, df = 17, P= 0.00325) and cholesterol (4.26+1.09 vs. 5.51+0.95 Student's T = 2.65, df = P = 0.016) were also lower in the SUD group, suggesting a possible nutritional contribution. It should also be noted that differences between the groups in serum iron levels disappeared on non-parametric testing (Wilcoxson's, Friedman and Sign tests all non-significant), and the Friedman and Sign test were non-significant for cholesterol alterations (Wilcoxson's T = 3.00, Z = 2.100, P = 0.036).

However the triglyceride and blood glucose levels were not different in the two groups (by all four tests).

### Haematological Values

**Figure 1:**
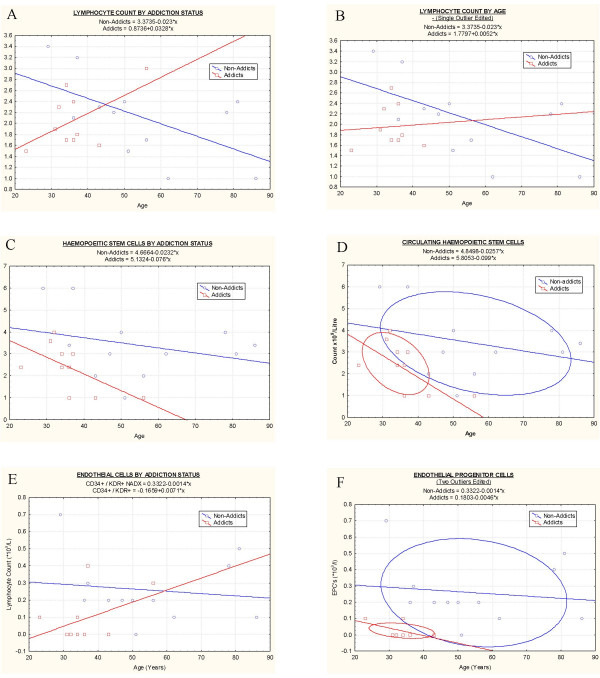
**Hematological parameters by age**.: A: Lymphocyte count; B: Lymphocyte Count, Single outlier excluded; C: Haemopoetic Stem Cells; D: Haemopoetic Stem Cells with ellipses at 95% confidence intervals ; E: Endothelial progenitor cells (EPC's); F: EPC's with two outliers excluded and C.I. Ellipses.

Haematological values are listed in Table 3 and illustrated graphically in Figure 1. Detailed information relating to ranges of these parameters is given in Table 4. As demonstrated in Table 3, there is no significant difference in most of these parameters. Data relating to the MCV and iron levels was mentioned above.

**Table 4:** Laboratory data ranges. Ranges: minimum-maximum

	**SUD**	**Non-SUD**
**Biochemistry**		
**ALT (IU/l)**	13–193	10–105
**Iron**	5–21	11–23
**Albumin**	38–46	41–49
**Cholesterol**	2.8–6.4	4.1–7.0
**Triglyceride**	0.6–2.5	0.5–2.1
**BSL**	3.8–11.1	3.5–6.0
		
**Haematology**		
**Haemoglobin**	121–157	126–166
**MCV**	82–90	88–98
**Platelets**	188–404	148–396
**WCC**	4.00–12.10	5.30–11.80
**Lymphocytes**	1.60–3.50	1.00–3.40
**Monocytes**	0.20–1.00	0.40–1.20
**Mononuclears**	1.80–4.50	0.00–4.40
**Neutrophils**	1.80–7.50	2.90–7.10
**CD34+CD45+ - HSC's**	1.00–4.00	1.00–6.00
**CD34+/KDR+ - EPC's**	0.00–0.40	0.00–0.70

Absolute lymphocyte (2.23+0.67 vs. 2.12 + 0.78 × 10^9^/l, Student's T = -0.35, df = 19, P = 0.73) and mononuclear (2.76+0.86 vs. 2.18+1.43 × 10^9^/l Student's T = -1.10, df = 19, P = 0.28) counts were similar (Table 3) as were their ranges (Table 4). Haemopoietic stem cells (HS's) were not different in the two groups (2.38+1.09 Vs. 3.40+4.56 cells/mcl, Student's T = 1.72, df = 19, P = 0.10). Endothelial progenitor cells (EPC's) were lower in the SUD (0.09+0.14 Vs. 0.26+0.20 cells/mcl, Student's T = 2.25, df = 19, P = 0.0367) both by absolute counts, and when numbers with EPC counts less than 0.15 × 10^11 ^were considered (Fisher Exact Test = 0.0299, Yates corrected Chi Squared 0.047, df = 1, O.R. = 0.09, C.I. 0.01–0.97) notwithstanding the significantly older age of the N-SUD group. The interpretation of these differences is, of course, complicated by the difference in ages between the two groups. Nevertheless, there is much published evidence that both HSC's and EPC's tend to decline with age[25]. The higher mean age of the control sample is therefore likely to result in an **under-estimate **of any effect of drug addiction on HSC's or EPC's.

Figure 1A shows the relationship of the lymphocyte counts with age in both addicted and non-addicted groups. When a linear model is fitted to these data (including SUD/N-SUD effects, a linear age effect, and a group by age interaction) the group by age interaction is not statistically significant. An approximate analysis was conducted, using a lowess[30] fit of EPC and HSC to age, for each group, and a common lowess fit to both groups. An approximate F statistic approached significance (P = 0.07), indicating weak evidence in favour of a different relationship between age and both HSC and EPC for the SUD and N-SUD groups – with the SUD group having lower numbers of both HSC's and EPC's compared with NA group members of equivalent age. This analysis is preliminary, and should be confirmed by further studies with larger sample sizes before the conclusion can be considered robust.

Figure 1B depicts similar data when a single outlier has been excluded. Confirmation that there is a different relationship in addicted and non-addicted patients' total lymphocyte counts with age has been submitted for publication in a large group of patients^31,^but the effect is quantitatively mild. Indeed in a group of 377 addicts and 2748 controls, the mean lymphocyte counts were 2.41(+0.03) and 2.26(+0.01), or only a 6.6% difference which does not alone explain the observed difference in progenitor cell counts. Figure 1C shows a clear separation of the HSC counts by age in the two groups which is emphasized when ellipses set at 95% confidence intervals are included (Figure 1D). Figure 1E shows similar data for EPC's with age, and Figure 1F shows the separation of the two groups shown by ellipse fitting at the C.I.'s. For both HSC's and EPC's there appeared to be a decline with age which was more marked in the addict group.

The significance testing of these various comparisons was little changed if non-parametric statistical tests (Friedman ANOVA, Wilcoxson matched pairs and Sign test) were applied to these studies. A detailed analysis for the bivariate Friedman ANOVA test is summarized in Table 5.

**Table 5:** Non-paramertic significance testing Friedman ANOVA results

	**N**	**df**	**Chi Square**	**Coefficient of cncordance**	**P**
**Age**	10	1	6.400	0.640	0.01141
**Height**	10	1	5.444	0.544	0.01963
**Weight**	10	1	0.400	0.400	0.52709
**Body Mass Index**	10	1	1.600	0.160	0.20590
**BP Systolic**	8	1	0.200	0.025	0.65472
**BP Diastolic**	8	1	1.000	0.125	0.31731
**Cholesterol**	8	1	2.000	0.250	0.15730
**Triglycerides**	8	1	0.000	0.000	1.00000
**BSL**	8	1	0.500	0.625	0.47950
**HCV**	10	1	7.000	0.700	0.00815
**ALT**	8	1	3.571	0.446	0.05878
**Iron**	8	1	1.285	0.160	0.25684
**Albumin**	8	1	4.500	0.562	0.03390
**Cigarettes**	10	1	8.000	0.800	0.00468
**Cigs +/-**	10	1	6.000	0.600	0.01431
**Alcohol**	10	1	3.000	0.272	0.83270
**Cannabis**	10	1	7.000	0.700	0.00815
**Amphetamine**	10	1	7.000	0.700	0.00815
**Heroin**	10	1	10.000	1.000	0.00157
**Opiate Years**	10	1	6.400	0.640	0.01141
**Haemoglobin**	10	1	6.400	0.640	0.01141
**MCV**	8	1	4.500	0.562	0.03390
**Platelets**	8	1	0.500	0.063	0.47950
**WCC**	8	1	2.000	0.250	0.15730
**Lymphocytes**	10	1	1.600	0.160	0.25090
**Monocytes**	8	1	0.000	0.000	1.00000
**Mononuclears**	10	1	1.600	0.160	0.20590
**Neutrophils**	8	1	0.500	0.063	0.47950
**HSC's**	10	1	0.500	0.050	0.47950

## Discussion

These data are significant as they are the first to our knowledge to interrogate the issue which is believed to be important in both the biology of ageing and addiction medicine in relation to the changes in circulating stem cell numbers which occur with age. They demonstrate a proof of principal effect that such changes can be studied in addicted patients and potentially interesting and important conclusions can be drawn. Having said that present data are admittedly preliminary only. Whilst they clearly invite further investigation in many different respects, it is important not to overstate the implications which can realistically be made from such an initial pilot report.

These data are notable because they suggest a clear separation between addicts and controls in both the general haemopoietic and the endothelial stem cell progenitors which achieves statistical significance in the case of the EPC's. In fact the degree of separation of the two groups is more marked than in any other dataset on addiction to our knowledge. Based on the data presented the decline in stem cells circulating in the peripheral blood appears to be three or four times as fast in addicts as in the general population. It also seems noteworthy to us that statistical significance has been reached with such small datasets. This fact together with the obvious separation of the two groups on graphical analysis suggests that the effect is both real and potentially important. Therefore our data, although clearly preliminary, are consistent with published data suggesting impaired cell growth and potentiation of apoptosis due to addictive drugs[1-3]. As noted in the presentation of the results some of our data (marginally lower iron, cholesterol and albumin in SUD) is perhaps consistent with a nutritional contribution to this effect; however the triglyceride and blood sugar data was not different between the two groups. Cholesterol and albumin are well known to have an hepatic synthetic contribution to their serum levels and alterations of liver function are well known in populations of SUD using drugs by the intravenous route (although they were not demonstrated in this study).

Should these early results be confirmed more generally by further studies with larger patients numbers and additional cell lines (including presently developmental neurogenesis imaging techniques[32-34]), these findings have very significant potential implications for understanding the cumulative toxicology of indefinite maintenance therapies and programmes both for opiate dependency, and, because other addictive drugs have similar cytostatic effects, the notion presently widespread in medicinal chemistry, clinical, research funding and other circles that agonist medication is functionally superior to sustain patient compliance in the long term to antagonists, for the whole direction of the development of the much needed adjuctive pharmacotherapies for other chemical dependencies. If confirmed the present results are likely to have far reaching implications for clinical practice and hence public policy, particularly as an increasing number of long acting depot preparations of antagonists are currently entering the marketplace[35,36]. Hence this preliminary study suggests further lines of investigation for on-going research in this important field, with significant future implications for drugs policy administrations and long term patient treatment development.

These findings achieve particular significance in the light of landmark papers suggesting remarkable prognostic significance particularly to the endothelial stem cell count as a predictor of cardiovascular risk and long term outcome[25]. This is underscored by the unusual morbidity and mortality well known to be associated with drug addiction, and strongly suggests that other mechanisms may be involved in the clinical features of addiction in addition to the usually described toxicities such as respiratory depression and co-occurring mental illness. They suggest also that, if this reduction represents in fact a generalized defect of cell renewal, it may be an important factor underlying the widely recognized elevated mortality of drug addiction, which in some series has been reported to be 10–70 times that of non-clinical populations[37,38]. Clinical evidence in relation to hair greying, that a generalized defect of pigmentary stem cells likely exists right across the scalp, was recently reported from this clinic[20].

The present dataset does not allow dissection of the major chemical – or for that matter lifestyle – factor/s which might be responsible for the observed dramatic deficit in circulating progenitor cells, but this is clearly an important point for further clinical and laboratory studies.

The techniques of flow cytometry lend themselves to the simultaneous testing of these concepts in multiple circulating stem and progenitor cell lines. In addiction to the two lines studied here, monoclonal antibodies directed against bone alkaline phosphatase and osteocalcin (for osteoblastic progenitors[39]), cytokeratin 5 and chemokine CXCR4 (for epithelial progenitors[40]) and oil red O staining (for adipogenic progenitors[41]) have been described. Indeed molecular parameters of ageing in such stem cells[42] can also be quantified by applying appropriate monoclonal antibodies in flow cytometric assays. The availability of techniques for stem and progenitor cells of many tissue specificities suggests that this technique lends itself to expansion of use with stem cells from multiple tissue lineages and formal quantitation of the pro-ageing hypothesis. Combination of flow cytometry with cell sorting will allow other techniques to be applied including tests of stem cell function including replicative limits, genetic and chromosomal integrity, susceptibility to oxidative stress, mitochondrial function and biogenesis, and molecular markers of ageing (including p16^INK4A^, p15^INK4B^, p16^ARF^, Bmi1, Dec1, Mcl1, DcR2, acidic β-galactosidase, telomere length and heterochromatin foci) [42-45]. Such studies are planned in our laboratories in the near future.

If addictive agents impair cell regeneration and potentiate cell death and senescence pathways in vivo as has been suggested in vitro, this carries obvious implications for the long term health impacts of SUD patients. If however, this regenerative defect occurs in the presence of addiction induced disordered DNA repair and elevated DNA replication error rates and major chromosomal translocation and non-disjunction errors as have been noted46, then with further investigations, the implications of addiction for cell regeneration both quantitatively and qualitatively, may prove to be profound indeed. Cancer is well known to be part of the spectrum of disorders which become increasingly common with age[11], and has also been described in addiction[47]. Many oncogenic molecular pathways exist in addiction[46,48-50]. Furthermore at the cellular level there are increasing pathways described between cellular senescence and growth arrest on the one hand and oncogenesis on the other[42,51]. If disruption of the integrity of DNA replication and repair mechanisms is demonstrated by relevant assays, the impact of disordered tissue regeneration as suggested by the present results would be amplified.

Furthermore it is important to note that these results also suggest interesting parallels between addiction and the ageing process which may well prove a fruitful line of investigation for both fields of bioscientific investigation.

This study carries significant proof of concept implications in several medical disciplines including addiction, the biology of ageing and clinical toxicology, and has public policy implications both for drugs policy administration and preferred patient treatments. That is to say that at the level of patient treatment, indefinite maintenance agonist treatment as is commonly advocated for opiate and other addictions may have to be re-considered; indeed the basic toxicology of addiction itself may have been significantly underestimated. Should such results be confirmed by larger scale more detailed investigations, such results invite a careful review of the evidentiary basis of the drugs policy debate. Better understanding of the molecular pathophysiological mechanisms responsible could improve our understanding of the biology of ageing, lead to new ways of investigating common age related disorders, and suggest new paths to treatment development. If cell regeneration in addiction is not just suppressed but disorganized this has further implications for expanding our understanding of addiction-related, heritable and other oncogenesis.
